# Antivenom Neutralization of Coagulopathic Snake Venom Toxins Assessed by Bioactivity Profiling Using Nanofractionation Analytics

**DOI:** 10.3390/toxins12010053

**Published:** 2020-01-16

**Authors:** Chunfang Xie, Julien Slagboom, Laura-Oana Albulescu, Ben Bruyneel, Kristina B. M. Still, Freek J. Vonk, Govert W. Somsen, Nicholas R. Casewell, Jeroen Kool

**Affiliations:** 1 Division of BioAnalytical Chemistry, Amsterdam Institute of Molecular and Life Sciences, Vrije Universiteit Amsterdam, De Boelelaan 1085, 1081 HV Amsterdam, The Netherlands; c.xie@vu.nl (C.X.); j.slagboom@vu.nl (J.S.); b.bruyneel@vu.nl (B.B.); k.b.m.still@vu.nl (K.B.M.S.); g.w.somsen@vu.nl (G.W.S.); 2 Centre for Analytical Sciences Amsterdam (CASA), 1098 XH Amsterdam, The Netherlands; 3 Centre for Snakebite Research & Interventions, Liverpool School of Tropical Medicine, Pembroke Place, Liverpool L3 5QA, UKNicholas.Casewell@lstmed.ac.uk (N.R.C.); 4 Centre for Drugs and Diagnostics, Liverpool School of Tropical Medicine, Liverpool L3 5QA, UK; 5 Naturalis Biodiversity Center, 2333 CR Leiden, The Netherlands; freek.vonk@naturalis.nl

**Keywords:** antivenom, nanofractionation, coagulation, anticoagulation, snakebite

## Abstract

Venomous snakebite is one of the world’s most lethal neglected tropical diseases. Animal-derived antivenoms are the only standardized specific therapies currently available for treating snakebite envenoming, but due to venom variation, often this treatment is not effective in counteracting all clinical symptoms caused by the multitude of injected toxins. In this study, the coagulopathic toxicities of venoms from the medically relevant snake species *Bothrops asper*, *Calloselasma rhodostoma*, *Deinagkistrodon acutus*, *Daboia russelii*, *Echis carinatus* and *Echis ocellatus* were assessed. The venoms were separated by liquid chromatography (LC) followed by nanofractionation and parallel mass spectrometry (MS). A recently developed high-throughput coagulation assay was employed to assess both the pro- and anticoagulant activity of separated venom toxins. The neutralization capacity of antivenoms on separated venom components was assessed and the coagulopathic venom peptides and enzymes that were either neutralized or remained active in the presence of antivenom were identified by correlating bioassay results with the MS data and with off-line generated proteomics data. The results showed that most snake venoms analyzed contained both procoagulants and anticoagulants. Most anticoagulants were identified as phospholipases A_2_s (PLA_2_s) and most procoagulants correlated with snake venom metalloproteinases (SVMPs) and serine proteases (SVSPs). This information can be used to better understand antivenom neutralization and can aid in the development of next-generation antivenom treatments.

## 1. Introduction

According to the World Health Organization around 5.5 million people get bitten by snakes, per year causing more than 1.8 envenomings, resulting in 81,000–138,000 deaths [1,2,3,4]. Furthermore, around 400,000 are estimated to suffer from long-term morbidity including amputation, blindness, and nerve damage [3,5,6,7,8].

Most snake venoms are abundant in peptides and proteins, many of which are of pathological importance and capable of affecting important physiological functions including blood coagulation, blood pressure regulation, and the transmission of nervous and muscular impulses. One of the most common but serious pathological effects of envenoming by snakes is coagulopathy [9], which is caused by the deregulation of blood coagulation by hemotoxic compounds [10]. Coagulopathy-inducing venom components can be categorized based on their procoagulant and anticoagulant properties. Important procoagulant toxins include certain clotting factor activating snake venom metalloproteinases and serine proteases [8,11,12], whereas anticoagulant toxins include enzymatic (e.g., phospholipases A_2_ (PLA_2_), fibrin(ogen)olytics and L-amino acid oxidases) and non-enzymatic proteins (e.g., C-type lectin-like proteins (CTL), three-finger toxins, and Kunitz-type proteinase inhibitors) [13]. They often synergistically disturb the hemostatic system and can lead to coagulopathy [8,9,14,15].

The only standardized specific treatment currently available for neutralizing the medically important effects of snake venom toxins is antivenom [8,9,12]. However, due to limited availability of antivenom, the need for cold storage, varying quality and specificity, and because of ontogenetic, individual and geographic inter-specific venom variation, antivenom administration often does not provide an adequately effective treatment. Frequently, inappropriate antivenoms are applied. Even when a suitable antivenom is administered in many cases, not all clinical symptoms are counteracted, i.e., not all toxins from the injected venom cocktails are (fully) neutralized. The limited paraspecific immunological cross-reactivity of each particular antivenom and the variation in snake venoms at every taxonomic level therefore requires many different antivenoms and/or polyvalent antivenoms to be available. Nevertheless, the proportion of antibodies specific to any particular venom in polyvalent antivenoms are relatively low, meaning that larger doses of these antivenoms are typically required to effect cure [12,16]. This not only results in higher treatment costs, but also increases the risk of adverse immunological reactions [12,17,18,19]. Therefore it is important to develop a new generation of antivenoms that are paraspecifically effective at much lower doses than currently used [12,20]. Although antivenomics [21] have been successfully used to evaluate which venom toxins can bind efficiently to antivenom, this technique does not reveal whether the binding of antivenom to venom components neutralizes their toxicity (which has to be assessed by one or several biological assays). New analytics that can assess the effectiveness of antivenoms in neutralizing the pathology induced by individual venom toxins would be a valuable complementary tool to antivenomics. Nanofractionation analytics is regarded as an effective method for screening complex crude venoms to rapidly identify and characterize separated venom toxins [22,23]. Such nanofractionation analytics include liquid chromatographic (LC) separation, UV absorbance, and mass spectrometric (MS) detection followed by a bioassay after collection of low-volume fractions of the column effluent at a resolution of 2 s to 10 s [22]. Among many existing assays available to assess coagulopathic nature of snake venoms, the assay developed recently by Still et al. [10] is a sensitive high throughput and low volume 384-well format assay, which can directly be combined with nanofractionation analytics. In the study of Still et al. several other coagulation assay formats are also discussed in detail.

In this study we present an integrated analytical approach providing a valuable complementary tool for antivenomics in antivenom research. We used the aforementioned, recently developed, high-throughput screening (HTS) coagulation assay [10] integrated into nanofractionation analytics to assess the coagulopathic properties of fractionated venom toxins following liquid chromatography. The profiles of fractionated venom toxins from a number of medically important snake species capable of causing coagulopathic effects were obtained after which the neutralizing potency of antivenoms against these activities was assessed. The species included in this study were *Bothrops asper*, *Calloselasma rhodostoma*, *Deinagkistrodon acutus*, *Daboia russelii*, *Echis carinatus* and *Echis ocellatus*. Moreover, the fractioned coagulopathic venom toxins were identified using mass spectrometry and proteomics data acquired by Slagboom et al. [24]. Finally, the cross-reactivity of antivenoms against venoms of closely related snake species was evaluated to assess their efficacy, or lack thereof, in the neutralization of these venom components. These results will broaden our knowledge on the relative efficacy and cross-reactivity of existing antivenom therapies and can guide the future development of next-generation antivenom treatments.

## 2. Results

In this study, a nanofractionation approach was used to evaluate antivenom efficacy in neutralizing the coagulopathic properties of individual venom proteins. Following LC separation, both the procoagulant and anticoagulant activities of venom fractions were assessed using a low volume HTS coagulation bioassay performed in 384-well plates. An assessment of the coagulation activity of each venom fraction in the presence of varying concentrations of homologous (i.e., species-specific) antivenom was then performed. Coagulopathic activity was correlated with the MS and proteomics data obtained in parallel to determine which types of venom components were neutralized and if so to what extent.

### 2.1. Correlation of Coagulopathic Toxins to Coagulant Peaks

**Figure 1:**
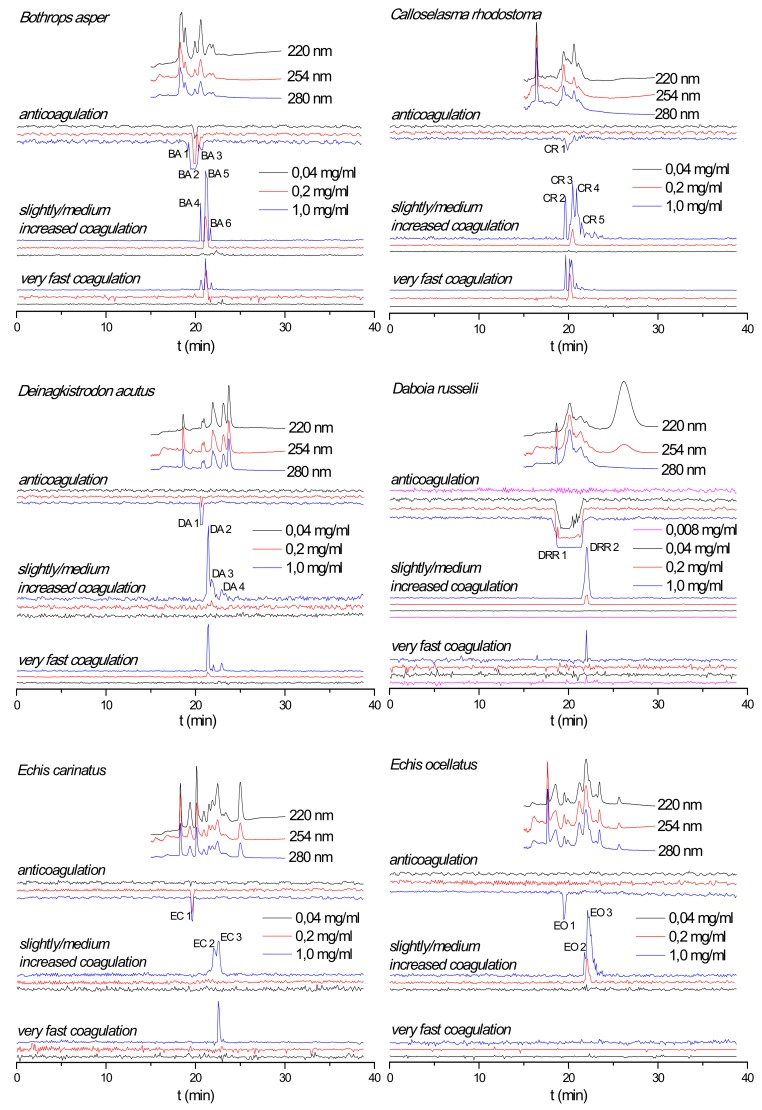
Reconstructed coagulation chromatograms for *B. asper*, *C. rhodostoma*, *D. acutus*, *D. russelii*, *E. carinatus*, and *E. ocellatus* venoms after nanofractionation at different concentrations. Numbers in the figures represent protein IDs and are listed in Table 1.

Coagulopathic venom components were correlated to coagulant peaks in each snake venom as described in the experimental section. The results are listed in Table 1. The table contains a numbered identifier for each positive or negative coagulopathic peak (these numbers are also given in Figure 1 where they indicate the respective peaks) together with Mascot results, accurate mass, toxin class, and activity. In multiple venom fractions, more than one venom toxin was assigned due to co-elution. Many snake venom PLA_2_s are known to exert anticoagulant activities [25,26]. Identified PLA_2_s that eluted in anticoagulant bioactivity regions are listed in Table 1 including those for which no data on their anticoagulation activity could be found in the literature and/or was reported in UniprotKB. The characteristic profiles of the UV chromatograms obtained for each venom were used to connect the results with MS and proteomics data obtained for the same venoms by Slagboom et al. [24]. This way, coagulopathic activities could be linked to accurate molecular masses and tentative protein identities. The exact mass data from LC-MS measurements and the proteomics results from the in-well tryptic digests of bioactive wells (performed by Slagboom et al. [24]) are presented in Table 1. Where no exact mass data could be acquired by LC-MS, the proteomics mass data obtained from the Mascot searches are provided.

### 2.2. Effect of Nanofractionated Venom Toxins on Plasma Coagulation

The effect of nanofractionated snake venom proteins on plasma coagulation was first studied in a dose-response manner. Reconstructed coagulation bioassay chromatograms are shown in Figure 1. For all venoms analyzed both procoagulant and anticoagulant effects were observed at a venom concentration of 1.0 mg/mL. The chromatographic retention times of the anticoagulants were within a similar time frame as those of the procoagulants, whereas the anticoagulants eluted closely together before the procoagulants. An exception was observed for *C. rhodostoma* venom for which the small anticoagulant peak (only observed at the highest venom concentration tested) eluted in between the cluster of procoagulant peaks. Some coagulopathic activities were observed as several sharp peaks as observed for *B. asper* venom, while other venoms showed only broad peaks in their chromatograms such as the anticoagulation activity of *D. russelii* venom. This broad anticoagulant peak most likely represents the bioactivity of multiple closely eluting peaks from several peptides and/or enzymes involved in the anticoagulant activity measured. As anticipated, when diluting injected venoms, all procoagulant and anticoagulant signals were concentration-dependent, i.e., both the height and broadness of the positive and negative peaks were reduced with decreasing venom concentrations until the signal disappeared. All coagulopathic signals in all tested venoms disappeared at a 0.04 mg/mL venom concentration, except for *D. russelii* venom, where the anticoagulant peak was still retained indicating full anticoagulant activity at this concentration. Only by further diluting this venom to 0.008 mg/mL we observed the disappearance of this potent anticoagulant peak. A detailed description of all observed coagulopathic peaks analyzed in duplicate for all venoms and their relative potencies are given in the Supporting Information (Section S1). Based on results from Slagboom et al. [24] the venom of the Australian elapid snake *Oxyuranus scutellatus* also displayed potent coagulopathic toxicity. Its effects on plasma coagulation and the neutralization efficacy of the corresponding Polyvalent Snake Antivenom (Australia-PNG) (CSL Limited, Parkville, Victoria Australia) against this venom are presented in the Supporting Information (Section S4).

### 2.3. Antivenom Neutralization Potency

**Figure 2:**
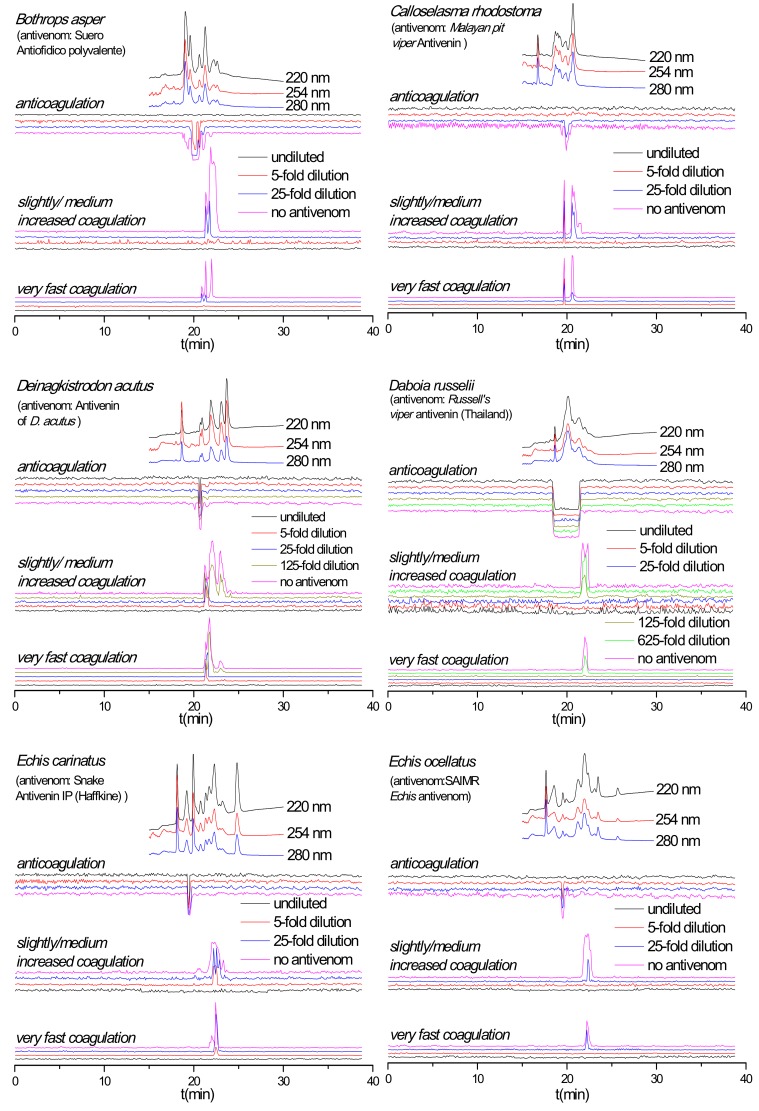
Coagulation chromatograms showing antivenom neutralization efficacy against nanofractionated venom proteins involved in modulating plasma coagulation. *B. asper*, *C. rhodostoma*, *D. acutus*, *D. russelii*, *E. carinatus* and *E. ocellatus* venoms analyzed in presence of different amounts/concentrations of antivenoms (i.e., no antivenom, x-fold dilution and undiluted antivenom).

**Figure 3:**
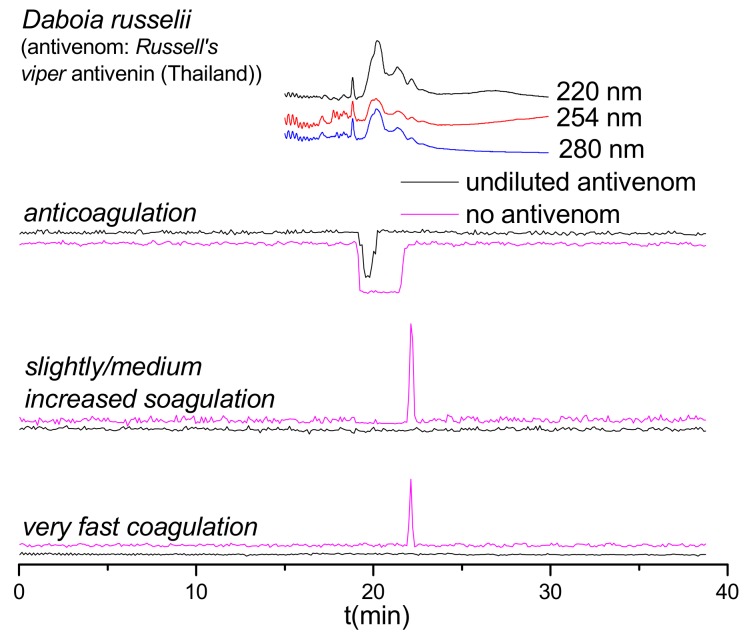
Coagulation chromatograms showing antivenom neutralization of venom proteins involved in modulating plasma coagulation. Nanofractionated *D. russelii* venom samples analyzed at 0.2 mg/mL (note: for all other antivenom analyses, 1.0 mg/mL venom concentrations were nanofractionated).

The capability of antivenoms to neutralize nanofractionated snake venom proteins involved in modulating plasma coagulation was studied at a venom concentration of 1.0 mg/mL. For all venoms the corresponding antivenom was analyzed at a minimum of three different concentrations, representing the normal clinically used antivenom concentration (undiluted), and the respective 5- and 25-fold antivenom dilutions. For some venoms, 125- and 625-fold antivenom dilutions were also evaluated (Figure 2). For most venoms, both the procoagulant and anticoagulant activities decreased with increasing antivenom concentrations. Specifically, when analyzed in the presence of undiluted antivenom, all coagulopathic signals were neutralized for *B. asper*, *C. rhodostoma* and *E. ocellatus* venoms. All procoagulant signals were fully neutralized for the *D. acutus*, *D. russelii* and *E. carinatus* venoms, whereas their anticoagulant activities were not fully neutralized or not neutralized at all. For the *D. acutus* and *E. carinatus* venoms, no neutralization of the single sharp anticoagulant peak was observed in their anticoagulation chromatograms at any of their respective antivenom concentrations. In case of *D. russelii* venom the anticoagulant neutralization (of the broad anticoagulant peak) did occur partly although this is not clearly visible in Figure 2, but when testing anticoagulation neutralization by undiluted antivenom using a 5-times lower *D. russelii* venom concentration, the neutralization was clearly visible (Figure 3). The coagulopathic activities neutralized by undiluted antivenom increased with decreasing antivenom concentrations. For both *B. asper* and *E. ocellatus* venoms, the procoagulant and anticoagulant peaks reappeared at the 25- and 5-fold antivenom dilutions, respectively. The procoagulant and anticoagulant activities in *C. rhodostoma* venom reappeared at the 5- and 25-fold antivenom dilutions, respectively. For *D. acutus*, *E. carinatus* and *D. russelii* venoms of which the procoagulant activities could be fully neutralized by their respective undiluted antivenoms, their procoagulant activity peaks started to appear at a 5-fold antivenom dilution for *D. acutus* and *E. carinatus* venoms, and at a 125-fold antivenom dilution for *D. russelii* venom. Finally, for the slightly/medium increased coagulation activities observed in the *D. acutus* venom chromatograms, the last two positive peaks were fully neutralized at a 25-fold antivenom dilution, while the first eluting positive peak was only efficiently neutralized by undiluted antivenom. The duplicate bioassay chromatograms and a detailed description of all coagulation activities neutralized by their corresponding antivenoms are given in the Supporting Information (Section S2).

As shown in Figure 2, the anticoagulant profile for *D. russelii* venom at 1.0 mg/mL could not even be neutralized by undiluted Russell’s viper antivenom. In fact, the antivenom did not neutralize any of the anticoagulant activities observed. Therefore, a lower venom concentration (0.2 mg/mL) was subsequently analyzed, both with and without undiluted Russell’s viper antivenom (Figure 3). Importantly, the Russell’s viper antivenom used here (Russell’s viper antivenom, Thai Red Cross) is purified from equine immunoglobulins after hyperimmunization with *D. siamensis* (Thailand) venom, while the venom used in this study is from the sister species *D. russelii* from Sri Lanka. The nanofractionated *D. russelii* venom displayed a sharp positive procoagulant peak and a broad negative anticoagulant peak at a concentration of 0.2 mg/mL, similar to those observed for the 1.0 mg/mL venom concentration, but slightly narrower, as expected. In the presence of undiluted antivenom, the procoagulant signals disappeared completely while the broad anticoagulant negative peak became narrower and smaller, indicating that most of the anticoagulant toxicities were neutralized by antivenom under these conditions.

### 2.4. Antivenom Cross-Reactivity

**Figure 4:**
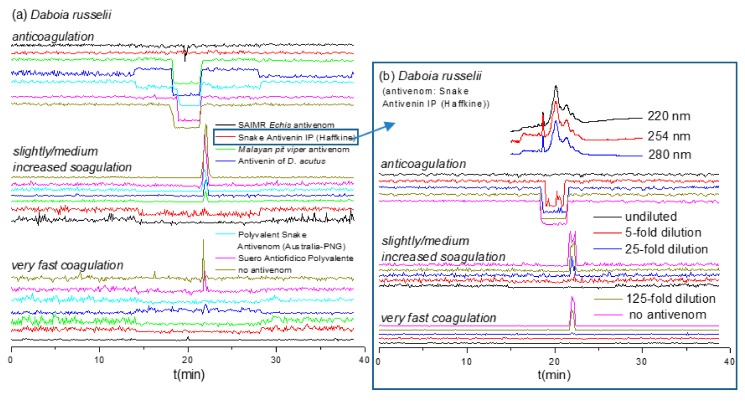
Coagulation chromatograms of antivenom cross-reactivity against nanofractionated *D. russelii* venom proteins: (**a**) The neutralization efficacy of various undiluted antivenoms against coagulopathic toxicities was assessed; (**b**) Snake Antivenin IP was selected and further analyzed at different antivenom concentrations.

While antivenom cross-reactivity for venoms of closely related snake species can perhaps be expected (although note our findings with *Daboia* spp. described above), more phylogenetically divergent venoms should display little to no cross-reactivity [27,28,29]. The venom of *D. russelii* showed potent (i.e., large and/or broad peaks) pro- and anticoagulant activities. Therefore, this venom was selected to evaluate the potential of our method for studying antivenom cross-reactivity, that is, evaluating antivenom potencies towards venoms other than their target venoms. Six undiluted antivenoms were evaluated against *D. russelii* venom after nanofractionation at a concentration of 1.0 mg/mL (Figure 4a). Our resulting data show that Snake Antivenin IP (Haffkine, India) and SAIMR *Echis* antivenom (SAVP, South Africa) can effectively neutralize the coagulopathic components of *D. russelii* venom. No coagulopathic activity was detected in the presence of undiluted Snake Antivenin IP and fast coagulation activity was readily neutralized by its 25-fold dilution (Figure 4b). These findings are perhaps not surprising when considering that this antivenom is made using Indian *D. russelii* venom as one of four immunogens. However, the procoagulant activities of *D. russelii* venom were also fully neutralized by undiluted SAIMR *Echis* antivenom, although a small sharp negative anticoagulant peak was still retained. In sharp contrast, “*D. acutus* Antivenin” (CDC, China) and “Suero Antiofidico Polyvalente” (ICP, Costa Rica) antivenoms could only partly neutralize the pro-coagulopathic activities observed in *D. russelii* venom, and most anticoagulant peaks were not neutralized. Moreover, both “Malayan pit viper antivenom” (Thai Red Cross, Thailand) and “Polyvalent Snake Antivenom” (CSL, Australia) could fully neutralize the fast procoagulant activity but could only partially reduce the slightly/medium increased procoagulant and anticoagulant activities.

**Figure 5:**
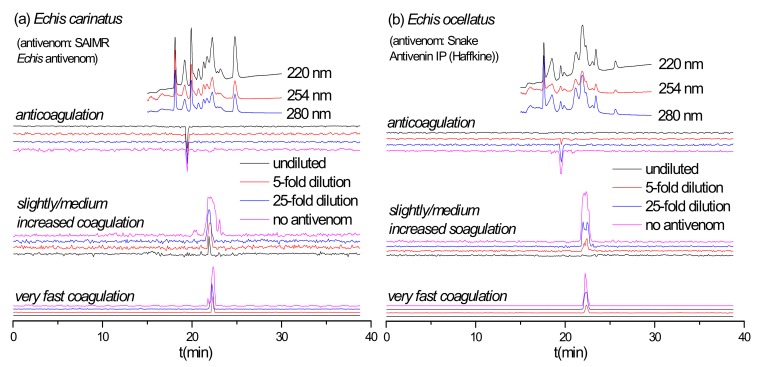
Coagulation chromatograms of antivenom cross-reactivity: (**a**) SAIMR *Echis* antivenom neutralization of nanofractionated *E. carinatus* venom toxins; (**b**) Snake Antivenin IP neutralization of nanofractionated *E. ocellatus* venom toxins.

Although venom composition similarities might be expected for *E. ocellatus* and *E. carinatus*, as they both belong to the *Echis* genus [30,31,32], studies have shown that anti-*E. ocellatus* antivenom is preclinically ineffective against the venom of *E. carinatus* [33], while clinical studies have shown that using anti-*E. carinatus* antivenoms for treating bites by *E. ocellatus* result in dramatic increases in patient case fatality rates [34]. To explore such complex interactions between these two medically important snake venoms and their antivenoms we analyzed the neutralization of *E. ocellatus* coagulotoxins by Snake Antivenin IP and that of *E. carinatus* venom by SAIMR *Echis* antivenom (anti-*E. ocellatus* and *E. pyramidum*) were analyzed (Figure 5). Perhaps surprisingly, undiluted Snake Antivenin IP fully neutralized the procoagulant and anticoagulant activities in *E. ocellatus* venom, with *E. ocellatus* venom being more effectively neutralized than *E. carinatus* venom. However, SAIMR *Echis* antivenom showed limited paraspecificity against the coagulopathic toxicity of *E. carinatus* venom and could only neutralize its fast procoagulant activity, even though it effectively neutralized all activities observed in the *E. ocellatus* venom. These findings suggest that toxins involved in causing other pathologies, such as hemorrhage for example, may be responsible for the described lack of clinical efficacy between Indian *E. carinatus* antivenom and snakebites by *E. ocellatus*. The duplicate chromatograms for SAIMR *Echis* antivenom vs. *E. carinatus*, Snake Antivenin IP vs. *E. ocellatus* and Snake Antivenin IP vs. *D. russelii* are presented in the Supplementary Materials (Section S3).

### 2.5. Limitations

As a critical note, although venom toxins are commonly highly stable and typically remain active when exposed to organic solvents used in reverse-phase LC, some might become (partly) denatured during separation and thereby lose their biological activity. This is perhaps most likely the case when considering relatively non-polar venom toxins eluting at higher concentrations of organic modifiers (i.e., acetonitrile). In contrary to LC-MS and venomics studies, in nanofractionation analytics the venom toxins have to survive the analytical separation non-denatured in order to perform post-column assays with the separated venom toxins. Additionally, the LC eluents have to be compatible with ESI-MS. The currently most efficient LC-MS separations of venoms available rely on trifluoroacetic acid (TFA) in the mobile phases for reversed-phase separations. The low pH resulting from the ion pairing agent TFA in the eluent, however, results in (partial or full) denaturation of certain toxins, particularly larger enzymes, including many pro-coagulopathic enzymes (data from preliminary studies conducted in our laboratory). Additionally, TFA in LC eluents is well known to reduce sensitivity and cause ion pairing observed in MS when performing LC-MS with directly coupled ESI-MS to the separation. Therefore, our reversed phase separations use formic acid (FA) instead when performing nanofractionation analytics. FA renders many venom toxin enzymes intact during separation but gives lower resolution and thus peak capacity resulting in more venom toxins being not fully separated from each other. As a consequence, for bioactive peaks in the bioactivity chromatograms, there are often closely eluting overlapping toxin candidates which can be responsible for the bioactivity observed. We are currently investigating different MS compatible separations and eluent compositions to get (1) high peak capacity, (2) eluted enzyme toxins in their native state and, (3) ESI-MS compatibility.

## 3. Discussion

Based on the data presented in Table 1 and Figure 2, Figure 3, Figure 4 and Figure 5 (which show antivenom neutralization of nanofractionated venom toxins), we assessed which coagulopathic venom protein(s) were efficiently neutralized, and which were less efficiently or not neutralized.

The *B. asper* anticoagulants (PA2H2_BOTAS and VM2_BOTAS) and procoagulants (VSPL_BOTAS, VM1B1_BOTAS and SLA_BOTAS) were neutralized by 25-fold dilutions and 5-fold dilutions of the species-relevant antivenom Suero Antiofidico polyvalente, respectively. Four *B. asper* PLA_2_s identified in Mascot searches (PA2H3_BOTAS, PA2HA_BOTAS, PA2B3_BOTAS and PA2A2_BOTAS) were matched to anticoagulant peaks, despite the fact that no anticoagulant activity has been reported for these enzymes in UniprotKB. However, many snake venom PLA_2_s are known to exert anticoagulant activities [25,26]. The anticoagulant activities of these PLA_2_s were neutralized but required doses of either undiluted or 25-fold dilution of Suero Antiofidico polyvalente antivenom. For *C. rhodostoma* three procoagulant (SLYA_CALRH, SLYB_CALRH and VSPF2_CALRH) and four anticoagulant (VSPF1_CALRH, SLEA_CALRH, SLEB_CALRH and PA2AB_CALRH) toxins were neutralized by a 25-fold dilution and 5-fold dilution of Malayan pit viper antivenom, respectively. The PA2BD_CALRH (PLA_2_) identified from Mascot searches was matched to anticoagulant peaks, displays calcium-independent myotoxicity [35] and was neutralized by a 5-fold dilution of *Malayan pit viper* antivenom. The PA2A_DEIAC and SL_DEIAC anticoagulants could not be neutralized by Antivenin of *D. acutus*. The VM11_DEIAC, VM1H5_DEIAC, VM3A2_DEIAC and VM3AH_DEIAC procoagulants, and the SLCB_DEIAC, VSP1_DEIAC, VSPA_DEIAC, VM1AC_DEIAC and VM3AK_DEIAC procoagulants were neutralized by a 25-fold dilution of and undiluted Antivenin of *D. acutus*. The PA2B8_DABRR, PA2B5_DABRR and PA2B3_DABRR PLA_2_s were identified as anticoagulants in *D. russelii* venom, and were neutralized by undiluted Snake Antivenin IP antivenom, undiluted SAIMR *Echis* antivenom and undiluted *Russell’s viper* antivenom, of which only PA2B8_DABRR was reported to exhibit anticoagulant activity [36]. The PA2A1_ECHCA (PLA_2_) was assigned as a tentative anticoagulant in the proteomics searches, was reported to be non-lethal to mice and devoid of neurotoxicity, myotoxicity, hemorrhage, anticoagulant activity and cytotoxicity [37], was not neutralized by either undiluted Snake Antivenin IP or SAIMR *Echis* antivenoms. The tentative anticoagulant PA2A5_ECHOC was neutralized by undiluted SAIMR *Echis* antivenom and undiluted Snake Antivenin IP. The VM3E2_ECHOC, VM3E6_ECHOC, SL1_ECHOC and SL124_ECHOC procoagulants were neutralized by a 5-fold dilution of SAIMR *Echis* antivenom and undiluted Snake Antivenin IP.

## 4. Conclusions

The neutralization potency of various antivenoms against the coagulopathic activities of (individual) venom toxins from several medically important snake venoms was assessed using a HTS coagulation assay following nanofractionation. When antivenoms were tested at their clinically used concentrations, all detected coagulopathic activities in *B. asper* and *C. rhodostoma* venoms were fully neutralized by their corresponding antivenoms. Antivenin of *D. acutus* was found to neutralize the procoagulant activities of the homologous venom, although the anticoagulant peaks were only partially neutralized. Surprisingly the Indian antivenom, Snake Antivenin IP (Haffkine), could fully neutralize coagulopathic toxins of both West African *E. ocellatus* and Indian *E. carinatus* venoms, with a higher potency against *E. ocellatus*. Conversely, the African SAIMR *Echis* antivenom was only capable of neutralizing the fast procoagulant activity of *E. carinatus* venom, but was effective against all coagulopathic activities exhibited by *E. ocellatus* venom. Most anticoagulant venom proteins in Sri Lankan *D. russelii* venom could only be neutralized by undiluted Russell’s viper antivenom (Thailand) when nanofractionated at a low (0.2 mg/mL), but not at the typically tested (1.0 mg/mL), concentrations. The Russell’s viper antivenom (Thailand) was, however, able to effectively neutralize all procoagulant toxicities caused by this venom. These findings suggest that considerable inter-specific differences exist between the anticoagulant toxins found in *D. russelii* and *D. siamensis* venoms. Almost all coagulopathic venom toxins in *D. russelii* venom were neutralized by the Snake Antivenin IP (Haffkine) antivenom, which includes Indian *D. russelii* venom as an immunogen, while the SAIMR *Echis* antivenom also exhibit some neutralizing potency against this paraspecific venom.

Thus, here we have demonstrated that by applying nanofractionation analytics, one can study the neutralization of separated venom toxins by antivenom instead of looking solely at crude venoms. As demonstrated in this study, the cross-reactivity of antivenoms against separated venom toxins can add great value in terms of informing antivenom neutralization capabilities in vitro, although careful interpretation of these findings must be applied, as the resulting neutralization profiles relate specifically to the bioassay being used, and careful consideration of the multifunctionality of snake venoms must be applied. Thus, neutralization of coagulopathic toxins may not necessarily mean that an antivenom will be clinically effective. However, by incorporating parallel conducted LC-MS and proteomics analyses (using data from Slagboom et al. [24]) we generated tentative identifications of the separated bioactive venom toxins thus further informing these neutralization profiles. The nanofractionation analytics described herein, therefore, serves as a valuable complementary antivenomics tool in antivenom research. Furthermore, such analytical approaches are also useful for studying environmental pollutants, plant extracts, and metabolic mixtures [38,39,40]. Ultimately, our data on antivenom neutralization can guide future research on the development of next generation antivenoms for treating snakebite. The cross-reactivity results are valuable for evaluating the potential use of antivenoms on distinct (but related) snake species and/or the same snake species from different geographic locations.

## 5. Materials and Methods

### 5.1. Chemicals

Water was purified using a Milli-Q Plus system from Millipore (Amsterdam, The Netherlands). Acetonitrile (ACN; LC/MS grade) and formic acid (FA) were obtained from Biosolve (Valkenswaard, The Netherlands). Calcium chloride (CaCl_2_) and phosphate-buffered saline (PBS) were purchased from Sigma-Aldrich (Zwijndrecht, The Netherlands). Bovine plasma (500 mL, Sodium Citrated, Sterile Filtered, Product Code: S0260) was purchased from Biowest (Nuaillé, France). Lyophilized snake venoms were stored at −20 °C. Prior to analysis, the snake venoms were dissolved in water at a concentration of 5.0 ± 0.1 mg/mL and then diluted in water to the defined concentrations in this study. After analysis, the samples were stored at −80 °C for further use. Antivenoms were stored at ~4 °C, according to the manufacturer’s instructions.

### 5.2. List of Venoms and Antivenoms Included in This Study

All venoms under study and their corresponding antivenoms were provided by the Centre for Snakebite Research and Interventions, Liverpool School of Tropical Medicine (Liverpool, United Kingdom). Suero Antiofidico polyvalente (Anti-Botropico, Anti-Crotalico, Anti-Laquesico) was obtained from the Clodomiro Picado Institute, University of Costa Rica (San Jose, Costa Rica) and was used as antivenom for *B. asper* (Costa Rica) venom. *Malayan pit viper* Antivenin from the Queen Saovabha Memorial Institute, The Thai Red Cross Society (Bangkok, Thailand) was used for neutralizing *C. rhodostoma* (Thailand) venom. Antivenin of *D. acutus* against *D. acutus* venom was sourced from the Center for Disease Control, NanKang (TaiPei, Taiwan). *Russell’s viper* antivenin (Thailand) obtained from the Queen Saovabha Memorial Institute, The Thai Red Cross Society (Bangkok, Thailand) was used to neutralize *D. russelii* (Sri Lanka) venom. SAIMR *Echis* antivenom used to neutralize *E. ocellatus* (Nigeria) was sourced from South African Vaccine Producers (PTY) Lid. (Edenvale, South Africa). Snake Antivenin IP (Haffkine) for neutralizing *E. carinatus* (India) venom was obtained from the Haffkine Bio-pharmaceutical Corporation Ltd. (Pimpri, India). Note that the Indian *E. carinatus* venom was collected from a single specimen that was inadvertently imported to the UK via a boat shipment of stone, and then rehoused at LSTM on the request of the UK Royal Society for the Prevention of Cruelty to Animals (RCPCA).

### 5.3. LC-MS Nanofractionation

Venom separation was performed on a Shimadzu UPLC system (‘s Hertogenbosch, The Netherlands) controlled by Shimadzu Lab Solutions software. For analysis, 50 μL venom solutions were injected by a Shimadzu SIL-30AC autosampler. The gradient separation was performed on a Waters XBridge reverse-phase C18 column (a 250 × 4.6 mm analytical column; 3.5 μm particle size diameter) at 30 °C. The column temperature was maintained by a Shimadzu CTO-30A column oven. The total solvent flow rate of 0.5 mL/min was controlled by two Shimadzu LC-30AD parallel pumps. Mobile phase A was made up of 98% H_2_O, 2% ACN and 0.1% FA and mobile phase B was 98% ACN, 2% H_2_O and 0.1% FA. The following LC gradient was used for separation: a linear increase from 0 to 50% B in 20 min followed by a linear increase from 50% to 90% B in 4 min followed by isocratic elution at 90% B for 5 min. Equilibration was done by a decrease from 90 to 0% B in 1 min followed by 10 min isocratic elution at 0% B. A 1 to 9 split ratio was implemented after the column of which the 10% fraction of the effluent was sent to a UV detector (Shimadzu SPD-M20A Prominence diode array detector) and the 90% fraction was directed to a Gilson 235P autoinjector modified into a nanofraction collector controlled by an in-house written software Ariadne, or to a FractioMate^TM^ nanofractionator (SPARK-Holland & VU, Netherlands, Emmen & Amsterdam) controlled by FractioMator software (Spark-Holland, The Netherlands, Emmen). The nanofractions were collected onto transparent 384-well plates (F-bottom, rounded square well, polystyrene, without lid, clear, non-sterile; Greiner Bio One, Alphen aan den Rijn, The Netherlands) at a resolution of 6 s/well. After fractionation, the plates with venom fractions were dried overnight using a Christ Rotational Vacuum Concentrator (RVC 2−33 CD plus, Zalm en Kipp, Breukelen, The Netherlands) in combination with a cooling trap at −80 °C. Freeze-dried plates were stored at −20 °C until bioassays were performed.

### 5.4. Plasma Coagulation Activity Assay

For aliquoting plasma from new bottles, a plasma bottle (500 mL bottle, Sodium Citrate, Sterile Filtered; Biowest, Nuaillé, France) stored at −80 °C was warmed up in a warm water bath until just fully defrosted and then quickly aliquoted in 15 mL CentriStar^TM^ tubes (Corning Science, Reynosa, Mexico), which were immediately stored at −80 °C. Prior to performing coagulation activity assays, aliquoted plasma was defrosted to room temperature in a warm water bath and then centrifuged at 2000 rpm for 4 min to remove possible particulate matter. The bioassay followed the method described recently by Still et al. [10]: A 20 mM CaCl_2_ solution (20 μL per well) at room temperature was pipetted onto a 384-well plate with freeze-dried nanofractionated venom fractions using a Multidrop™ 384 Reagent Dispenser (Thermo Fisher Scientific, Ermelo, The Netherlands). Then, plasma was pipetted into the plate using the same dispenser system (20 μL/well). In the first set of experiments, only varying concentrations of nanofractionated venoms were analyzed for their coagulopathic effects. For these experiments, where no antivenom was added to the incubations, the final assay volume was 40 μL/well. Immediately after plasma addition, a kinetic absorbance measurement was performed on a Varioskan™ Flash Multimode Reader (Thermo Fisher Scientific, Ermelo, The Netherlands) at a wavelength of 595 nm at 25 °C. The coagulation activity in each well was measured kinetically over 100 min. The resulting coagulation curves were normalized by dividing each slope value by the median of all the values obtained in a single measurement and plotted in several different graphs, that is, the slope of the average of the 0–5 min readings for fast coagulation, the slope of the average of the 0–20 min readings for slightly/medium increased coagulation, and the slope of the single reading at 100 min for anticoagulation. Finally, for each coagulopathic parameter (anticoagulation, fast coagulation, and extremely fast coagulation) the processed values per well were plotted against the chromatographic time of each fraction to generate bioactivity chromatograms. Procoagulant activity was plotted in two different ways to distinguish the slightly and medium increased coagulation velocity from the fast coagulation velocity. In this study, fast coagulation is considered full coagulation, resulting in maximum absorbance in the bioassay within 5 min. By plotting the slope of the readings from 0 to 5 min only, the slightly or medium increased coagulation velocity is not observed, as significant clotting is not yet observed. By plotting the slope of the readings from 0 to 20 min, all coagulation velocities reaching full coagulation before 20 min display the same slope and therefore their real coagulation velocities cannot be distinguished from each other.

To analyze the effect of antivenom on nanofractionated venom components, antivenom (10 µL; prepared as different dilutions from the clinically used antivenom solutions) was added to all wells of the 384-well plates containing freeze-dried nanofractionated venom. The diluted antivenom solutions were diluted 5, 25, 125 and 625-fold in PBS from the normal clinical concentration. Next, each plate was centrifuged for 1 min at 2000 rpm using a 5810 R centrifuge (Eppendorf, Germany) and then pre-incubated for 30 min at room temperature. Following this incubation period, the calcium and plasma solutions were added to the plates as described above, and then the plates were measured on the platereader. Each fraction was analyzed at least in duplicate. For comparison, to venom-only plates (without any antivenom added), 10 µL PBS was added to each well and pre-incubated in the same manner as done for the plates where antivenom was added.

### 5.5. Correlation of Coagulation Data with MS and Proteomics Data

For each procoagulant and anticoagulant peak observed in the bioactivity chromatogram, the corresponding accurate mass (es) was extracted from the MS chromatogram acquired in parallel. In addition, the proteomics data were also available from recent work in our group [24]. To import the proteomics data for the venoms included in this study, the fingerprint profile LC-UV traces (measured at 220 nm, 254 nm and 280 nm) for the same venom analyses acquired in both studies were used to align the chromatograms to each other by inspecting potential slight retention times differences followed by aligning one of the two chromatograms to the other when needed. Then the coagulation bioassay activities from this study could be correlated to the MS and proteomics data acquired from the same eluting venom toxins analyzed by Slagboom et al. [24]. Specifically, the UV data, coagulation bioassay data, MS-Total Ion Current (TIC) and Extracted Ion Current (XIC) data in the chromatographic profiles from Slagboom et al. were exactly aligned to those in our study, thus allowing for the comparison of all datasets and the matching of corresponding accurate masses and proteomics-based venom toxin identifications with each coagulation and anticoagulation peak. In this way, accurate masses and proteomics-based venom toxin identifications were obtained for most coagulation and anticoagulation peaks. The UniprotKB database was used to search for information on toxin class and potentially known functions of the relevant toxins.

## Figures and Tables

**Table 1:** Correlated LC-UV peaks, LC-MS (mass spectrometry) masses and proteomics data for coagulopathic venom toxins (peaks numbers of the pro- and anticoagulant peaks are indicated in Figure 1; CTL = C-Type Lectin; PLA_2_ = Phospholipases A_2_; SVMP = Snake Venom Metalloproteinase; SVSP = Snake Venom Serine Protease.

Species	Peak Number	Retention Time (min)	Mascot Protein Hits	Exact Mass From MS Data	Exact Mass Calculated From Mascot Data	Toxin Class
*B. asper*	BA 1	19.1	PA2H2_BOTAS	13,714.5646	13,714.56817	PLA_2_
	BA 2	19.4–20.2	PA2HA_BOTAS	13,912.4649	13,896.51308	PLA_2_
	BA 2	19.4–20.2	PA2H3_BOTAS	13,765.5812	13,765.58896	PLA_2_
	BA 3	20.5–20.8	PA2B3_BOTAS	13,957.5333	13,957.48720	PLA_2_
	BA 3	20.5–20.8	VM2_BOTAS	-	53,564	SVMP
	BA 3	20.5–20.8	PA2A2_BOTAS	-	14,194	PLA_2_
	BA 4	20.6	VSPL_BOTAS	-	28,019	SVSP
	BA 4	20.6	VM1B1_BOTAS	-	45,936	SVMP
	BA 5	21.1–21.3	SLA_BOTAS	-	7084	CTL
	BA 6	21.7	-	-	-	-
*C. rhodostoma*	CR 1	19.7–20.5	PA2BD_CALRH	13,665.0848	13,665.0237	PLA_2_
	CR 1	19.7–20.5	VSPF1_CALRH	-	26,570	SVSP
	CR 1	19.7–20.5	SLEA_CALRH	-	15,962	CTL
	CR 1	19.7–20.5	SLEB_CALRH	-	15,190	CTL
	CR 1	19.7–20.5	PA2AB_CALRH	-	14,352	PLA_2_
	CR 2	19.6–19.7	VSPF2_CALRH	-	29,145	SVSP
	CR 3	20.1–20.7	VSPF2_CALRH	-	29,145	SVSP
	CR 4	20.8–21.2	VSPF2_CALRH	-	29,145	SVSP
	CR 4	20.8–21.2	SLYA_CALRH	-	15,796	CTL
	CR 5	21.5–21.7	SLYA_CALRH	-	15,796	CTL
	CR 5	21.5–21.7	SLYB_CALRH	-	16,770	CTL
*D. acutus*	DA 1	20.6–20.9	PA2A_DEIAC	-	14,820	PLA_2_
	DA 1	20.6–20.9	SL_DEIAC	-	18,332	CTL
	DA 2	21.3–21.7	SLCB_DEIAC	-	17,133	CTL
	DA 2	21.3–21.7	VSP1_DEIAC	-	29,480	SVSP
	DA 2	21.3–21.7	VSPA_DEIAC	-	26,132	SVSP
	DA 2	21.3–21.7	VM1AC_DEIAC	-	47,690	SVMP
	DA 2	21.3–21.7	VM11_DEIAC	-	47,845	SVMP
	DA 2	21.3–21.7	VM1H5_DEIAC	-	46,518	SVMP
	DA 2	21.3–21.7	VM3AK_DEIAC	-	69,752	SVMP
	DA 3	21.8–22.1	VM11_DEIAC		47,845	SVSP
	DA 3	21.8–22.1	VM1H5_DEIAC		46,518	SVSP
	DA 4	22.8–23.1	VM3A2_DEIAC		27,151	SVMP
	DA 4	22.8–23.1	VM3AH_DEIAC		70,721	SVMP
*D. russelii*	DRR 1	18.3–21.7	PA2B8_DABRR	13,587.2248	13,587.2027	PLA_2_
	DRR 1	18.3–21.7	PA2B5_DABRR		13,587	PLA_2_
	DRR 1	18.3–21.7	PA2B3_DABRR		13,687	PLA_2_
	DRR 2	21.6–22.4	-	-	-	-
*E. carinatus*	EC 1	19.6–19.7	PA2A1_ECHCA	-	16,310	PLA_2_
	EC 2	21.9–22.3	-	-	-	-
	EC 3	22.3–22.9	-	-	-	-
*E. ocellatus* (Nigeria)	EO 1	19.4–19.8	PA2A5_ECHOC	13,856.1382	13,856.0665	PLA_2_
	EO 2	21.8–21.9	VM3E2_ECHOC	-	69,426	SVMP
	EO 2	21.8–21.9	VM3E6_ECHOC	-	57,658	SVMP
	EO 2	21.8–21.9	SL1_ECHOC	-	16,601	CTL
	EO 2	21.8–21.9	SL124_ECHOC	-	16,882	CTL
	EO 3	22.0–23.1	VM3E6_ECHOC	-	57,658	SVMP
	EO 3	22.0–23.1	SL1_ECHOC	-	16,601	CTL
	EO 3	22.0–23.1	SL124_ECHOC	-	16,882	CTL

## Supplementary Materials

The following are available online at https://www.mdpi.com/2072-6651/12/1/53/s1, Figure S1: Reconstructed duplicate coagulation bioassay chromatograms for *B. asper*, *C. rhodostoma*, *D. acutus*, *D. russelii*, *E. carinatus* and *E. ocellatus* venoms following nanofractionation, analyzed at different concentrations, Figure S2: Duplicate chromatograms for antivenom neutralization of nanofractionated venom proteins involved in modulating plasma coagulation for *B. asper*, *C. rhodostoma*, *D. acutus*, *D. russelii*, *E. carinatus* and *E. ocellatus* venoms, Figure S3: Duplicate chromatograms of 0.2 mg/mL nanofractionated *D. russelii* venom showing antivenom neutralization of venom proteins involved in modulating plasma coagulation (note: for all other antivenom experiments, 1.0 mg/mL venom concentrations were nanofractionated), Figure S4: Duplicate bioassay chromatograms of evaluating antivenom cross-reactivity in neutralizing venom components involved in modulating plasma coagulation for SAIMR *Echis* antivenom vs. *E. carinatus*, Snake Antivenin IP vs. *E. ocellatus* and Snake Antivenin IP vs. *D. russelii*, Figure S5: Reconstructed duplicate coagulation bioassay chromatograms of the Elapid venom of *O. scutellatus* after nanofractionation, analyzed at different concentrations, Figure S6: Duplicate bioassay chromatograms showing antivenom neutralization of nanofractionated *O. scutellatus* venom proteins involved in modulating plasma coagulation.
