# Supplementary material for: Antivenom Neutralization of Coagulopathic Snake Venom Toxins Assessed by Bioactivity Profiling Using Nanofractionation Analytics
Source: Toxins (Basel). 2020 Jan 16;12(1):53. doi: 10.3390/toxins12010053 (PMC7020444; doi:10.3390/toxins12010053)
Supplement: Supplementary file 1 [file toxins-12-00053-s001.pdf]

Supplementary Materials: Antivenom Neutralization of Coagulopathic Snake Venom Toxins Assessed by Bioactivity Profiling Using Nanofractionation Analytics

Chunfang Xie, Julien Slagboom, Laura-Oana Albulescu, Ben Bruyneel, Kristina B. M. Still, Freek J. Vonk, Govert W. Somsen, Nicholas R. Casewell and Jeroen Kool

S1. Effect of nanofractionated venom toxins on plasma coagulation

The reconstructed coagulation bioassay chromatograms showing the effect of individual venom proteins for *Bothrops asper, Calloselasma rhodostoma, Deinagkistrodon acutus, Daboia russelii* (Sri Lanka), *Echis carinatus* (India) and *Echis ocellatus* (Nigeria) venoms after nanofractionation at different concentrations are shown in Figure S1. In this study, each coagulation bioassay was performed at least in duplicate following nanofractionation for assessing reproducibility. In each Figure, the bioassay chromatograms depicted on the right which do not have superimposed correlated UV data represent replicates of the chromatograms on the left. For each venom, the lower series of superimposed bioassay chromatograms represent the analyses of fast coagulation for different venom concentrations (1.0 mg/ml being the highest concentration tested). The middle set represents slightly/medium increased coagulation and the upper series represents anticoagulation.

When the venoms were analyzed at 1.0 mg/ml, strong positive fast and slightly/medium procoagulant peaks were observed for *B. asper* venom, in addition to a clear (full anticoagulation) broad negative peak. This broad negative peak most probably represents the bioactivity of multiple closely eluting peaks from several peptides and/or enzymes involved in this activity. For both *C. rhodostoma* and *D. acutus* venoms, strong procoagulant (positive) and moderate anticoagulant (negative) peaks were observed. For *D. russelii* venom, a strong and broad anticoagulant activity peak, together with an extensive slightly/medium procoagulant and a moderate fast procoagulant peak were observed. *E. carinatus* venom exhibited a similar pattern to *B. asper* venom, as strong but sharp peaks were observed in the fast procoagulant, the slightly/medium procoagulant, and the anticoagulant chromatograms. *E. ocellatus* venom did not display fast procoagulant activity, but exhibited significant activity in both the slightly/medium procoagulant and the anticoagulant chromatograms.

Following the dilution of injected venoms, all procoagulant and anticoagulant signals decreased until all signals disappeared. For *B. asper* venom, at a venom concentration of 0.2 mg/ml, only a sharp positive peak was observed for both the fast procoagulant and the slightly/medium procoagulant activity, while a broad negative peak was still apparent at this concentration for the anticoagulant activity, despite being much narrower than that observed at 1.0 mg/ml. At a 0.04 mg/ml venom concentration, no procoagulant activity was retained, and only a small fraction of the initial anticoagulant activity was still observed as a sharp negative peak. The *C. rhodostoma* and *E. ocellatus* venoms displayed anticoagulant activity only at the highest concentration (1.0 mg/ml; 50 µl) and no anticoagulant activity was observed after further dilution (at 0.2 mg/ml and 0.04 mg/ml). With regards to procoagulant activity, two broad fast procoagulant and slightly/medium procoagulant peaks were observed at 1.0 mg/ml for *C. rhodostoma*, which narrowed to one sharp positive peak upon dilution, implying that one or several very closely eluting procoagulants are responsible for the majority of procoagulant effects observed in this venom; for *E. ocellatus*, the slightly/medium procoagulant activity disappeared at 0.04 mg/ml, while the fast procoagulant activity was not detected for any of the analyzed venom concentrations. The sharp and weak anticoagulant activity observed for *D. acutus* venom was only observed at the 1.0 and the 0.2 mg/ml venom concentrations, disappearing after further dilution to 0.04 mg/ml. No slightly/medium procoagulant peaks were observed after further dilution. A weak but sharp positive peak was noted at 0.2 mg/ml for fast procoagulation, and no activity was observed at the 0.04 mg/ml venom concentration. The *E. carinatus* venom showed a similar anticoagulant activity

profile as *E. ocellatus* venom at the highest concentration, and the activity was also observed at a 0.2 mg/ml venom concentration, but disappeared at 0.04 mg/ml. All procoagulant peaks in *E. carinatus* disappeared at a venom concentration of 0.2 mg/ml. The remarkably strong and broad anticoagulant activity (negative) peak observed in *D. russelii* venom at 1.0 mg/ml remained roughly unchanged at 0.2 mg/ml, and only at a 25-fold venom dilution (0.04 mg/ml) did the anticoagulant peak become narrower, yet still retained full anticoagulant activity, which was lost at 0.008 mg/ml. However, no fast procoagulant activity was observed at 0.2 mg/ml, while a weak but sharp slightly/medium procoagulant (positive) peak was retained. This positive peak disappeared in subsequent dilutions.

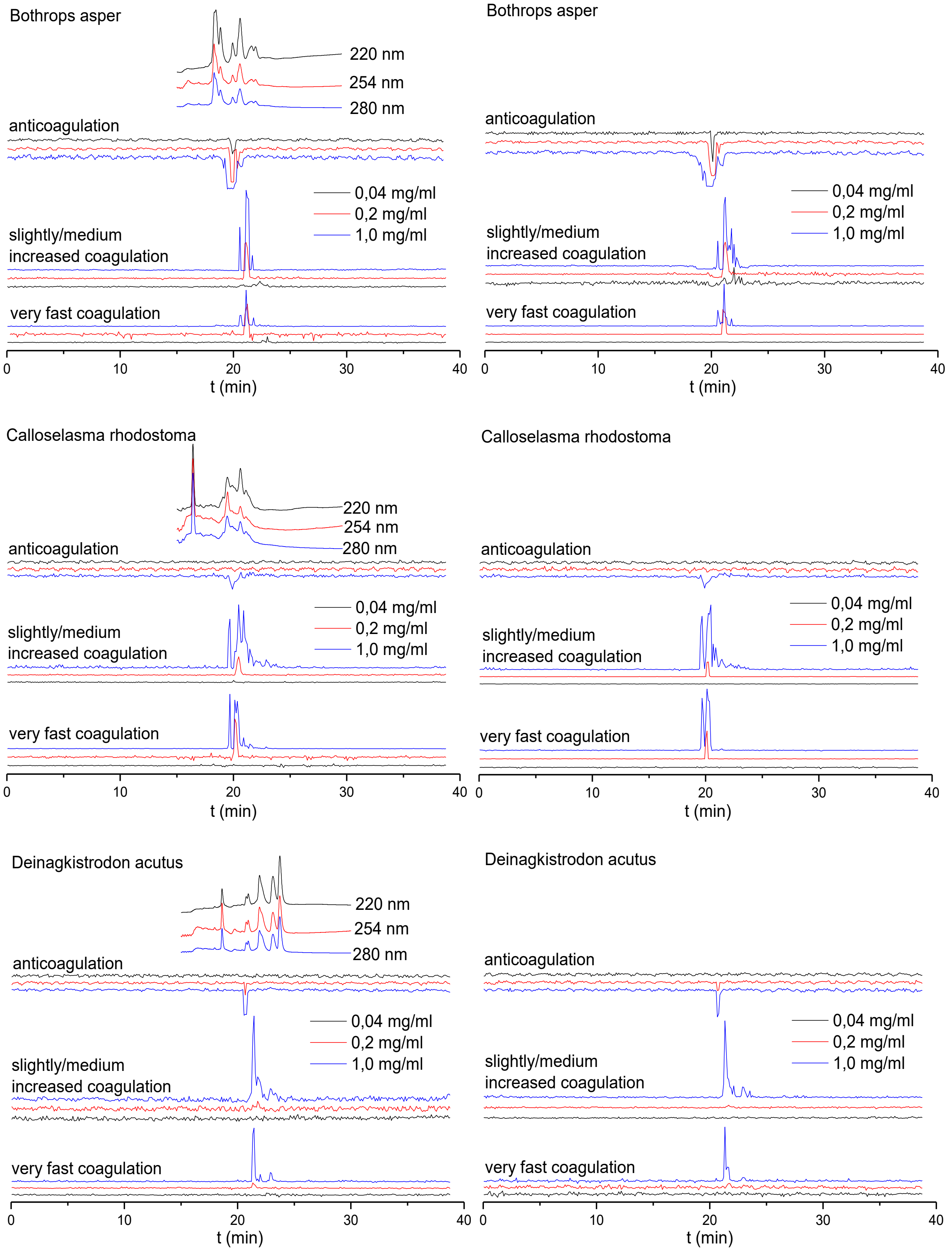

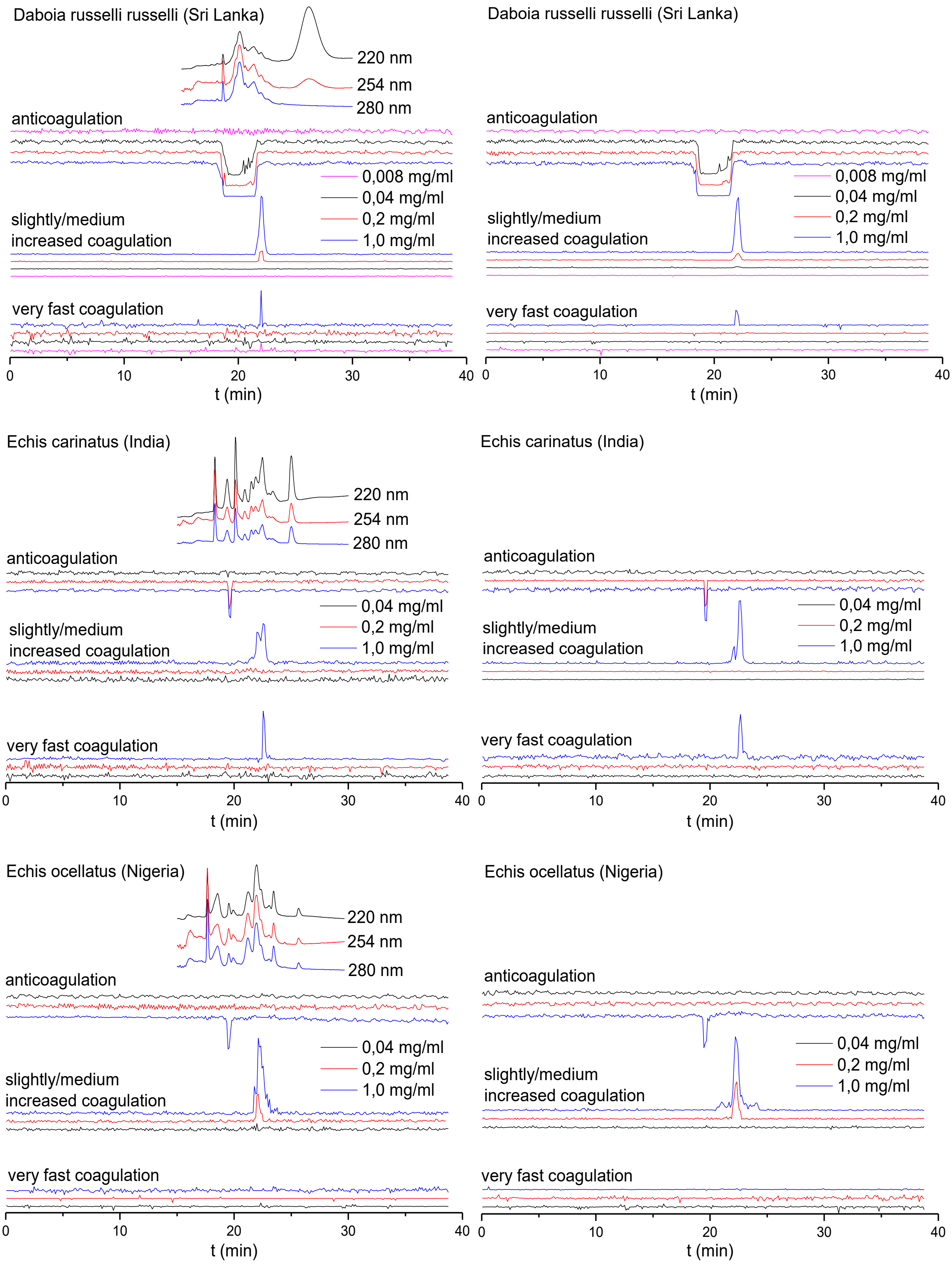

Figure S1. Reconstructed duplicate coagulation bioassay chromatograms for *B. asper*, *C. rhodostoma*, *D. acutus*, *D. russelii*, *E. carinatus* and *E. ocellatus* venoms following nanofractionation, analyzed at different concentrations.

S2. Antivenom neutralization potency

Reconstructed coagulation bioassay chromatograms showing antivenom neutralization potencies against nanofractionated venom proteins involved in modulating plasma coagulation for *B. asper*, *C. rhodostoma*, *D. acutus*, *D. russelii*, *E. carinatus* and *E. ocellatus* venoms are depicted in Figure S2. Venoms

were nanofractionation at a concentration of 1.0 mg/ml for all analyses. For *B. asper* venom, in the venom-only chromatogram (10 µl PBS solution was added before pre-incubation and is shown as "no antivenom" in the graphs), three sharp positive peaks were observed for the fast coagulation activity profile. One intense sharp positive peak followed by a relative broad intense positive peak were detected for the slightly/medium increased coagulation activity. A sharp negative peak followed by a broad negative peak and another sharp negative peak were observed in the anticoagulation activity chromatogram. For the undiluted antivenom trace, all the signals observed in the venom-only sample disappeared, indicating that all coagulopathic activities were neutralized by the antivenom. For the 5-fold antivenom dilution, the procoagulant fractions did not show any activity peaks. In the anticoagulation chromatogram however, the broad negative peak appeared as a sharp and weak negative peak, while the two sharp negative peaks were not observed. By further decreasing the antivenom concentration to a 25-fold dilution, two positive peaks were retained in both the fast coagulation and the slightly/medium increased coagulation activity chromatograms, while the third positive peak remained fully neutralized. Furthermore, in the anticoagulation chromatogram, the first sharp negative peak was still fully neutralized, and only the broad negative peak, followed by a sharp negative peak, were observed. For *C. rhodostoma* venom, two positive peaks were observed for the fast coagulation activity and three positive peaks for the slightly/medium increased coagulation activity in the venom-only analysis; no activity was detected for the undiluted antivenom trace, as all the detected coagulopathic toxicity peaks were fully neutralized. Only the first eluted positive peak could be observed at a 5-fold antivenom dilution analysis both in fast coagulation chromatograms and in slightly/medium increased coagulation chromatograms, while all other positive peaks were fully neutralized. For the 25-fold antivenom dilution, the two fast procoagulant peaks appeared weaker and sharper than those in the venom-only chromatogram; two positive peaks were now detected for the slightly/medium increased coagulation activity, while the third positive peak was still fully neutralized. Furthermore, for the anticoagulation activity, two negative peaks were observed in the venom-only trace and both activities were fully neutralized by undiluted or 5-fold diluted antivenom. The weak negative peak observed in the venom-only analysis was still neutralized by a 25-fold antivenom dilution, while the strong negative peak was retained. For *D. acutus* venom, in the case of the undiluted concentration antivenom analysis, all procoagulant activities were neutralized. Many positive peaks representing procoagulant activity were fully neutralized both at the 5- and 25-fold antivenom dilutions. One positive peak decreased with increasing antivenom concentrations in a concentration-dependent manner. In the 125-fold antivenom dilution profile, procoagulant signals were observed, but were weaker compared to the venom-only analysis. In contrast to the efficient neutralization of the procoagulant peaks, the sharp but significant negative signal representing anticoagulant proteins could not even be neutralized by undiluted antivenom. Only the first weak negative peak present in the venom-only trace disappeared at the 125-fold antivenom dilution, while the third negative peak in venom-only chromatogram disappeared only following incubation with undiluted antivenom. For *D. russelii* venom, the venom-only trace displayed a positive peak in the fast coagulation chromatogram and an intense positive peak in the slightly/medium increased activity chromatogram. A broad and strong negative activity peak was observed in the anticoagulation chromatogram. The procoagulant signals were fully neutralized by undiluted antivenom while the extensive and strong anticoagulant activity was not neutralized at all. By decreasing the antivenom concentration, the fast coagulation activity was neutralized up to the 625-fold antivenom dilution, while the slightly/medium increased coagulation activity was apparent at the 125-fold antivenom dilution. For *E. carinatus* venom, the sharp anticoagulant activity was not neutralized, while the procoagulant activity was fully neutralized by undiluted antivenom. There were two positive peaks indicating fast coagulation activity the weak peak was neutralized by the 5- and 25-fold antivenom dilutions, while the strong positive peak was not. A weak positive peak followed by a strong and broad positive peak and a sharp positive peak were detected in the venom-only trace in the slightly/medium increased coagulation chromatogram; the front weak and the tailing sharp positive peaks were readily neutralized by a 25-fold antivenom dilution, while the broad positive peak was retained and split into three sharp positive peaks with a lower total intensity than that measured in the venom-only analysis; of these three sharp positive peaks, only one was observed when incubating with a 5-fold antivenom dilution. For *E. ocellatus* venom, all

procoagulant and anticoagulant toxicities were fully neutralized by undiluted antivenom. The procoagulant toxicities were also fully neutralized by a 5-fold antivenom dilution, whereas the anticoagulant toxicities were only partly neutralized. For the 25-fold antivenom dilution analysis, the slightly/medium increased coagulation activity neutralized most coagulopathic toxicities, while the fast coagulation and the anticoagulation chromatograms displayed a slight decrease in total activity when compared to that in the venom-only analysis.

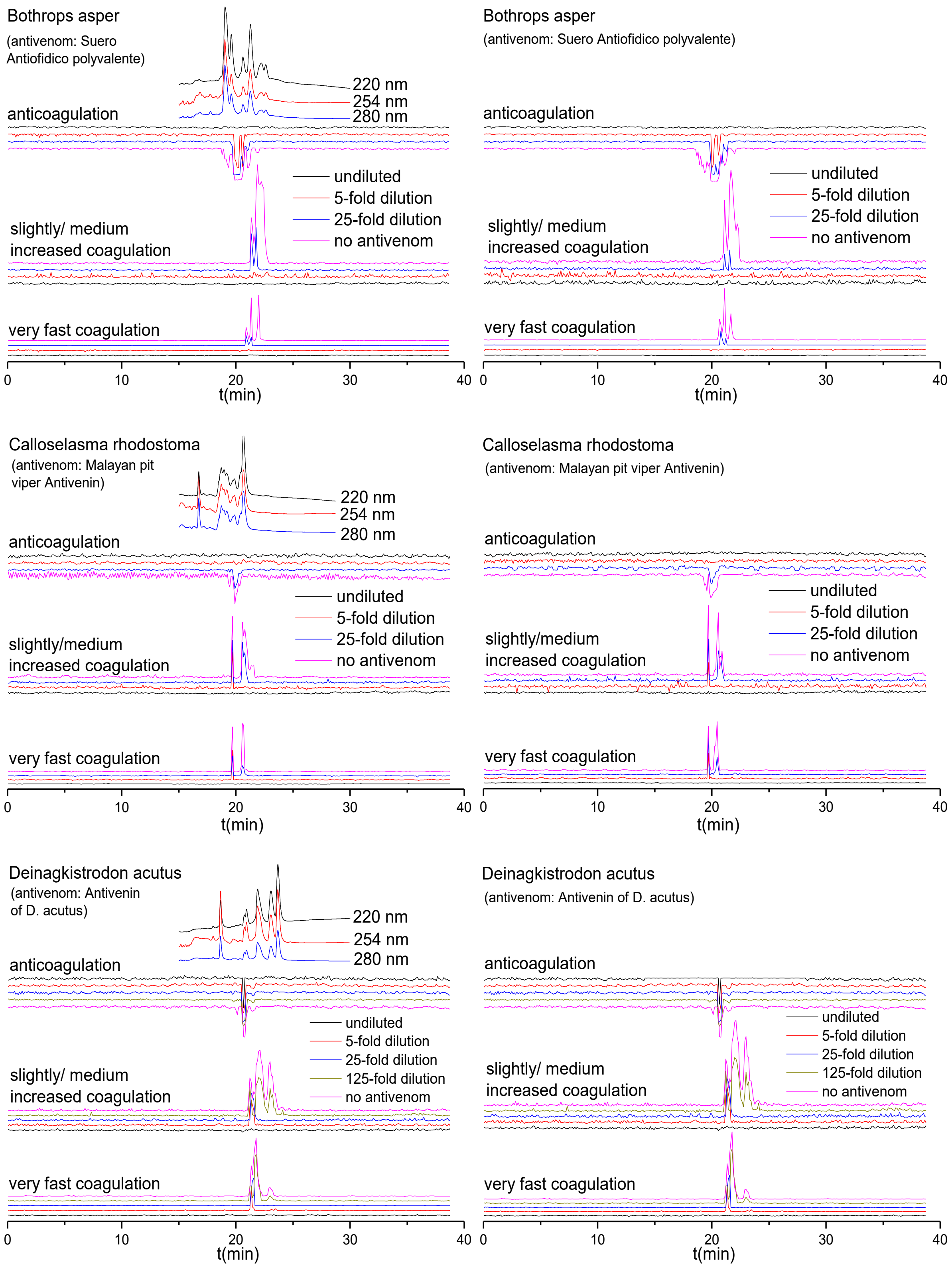

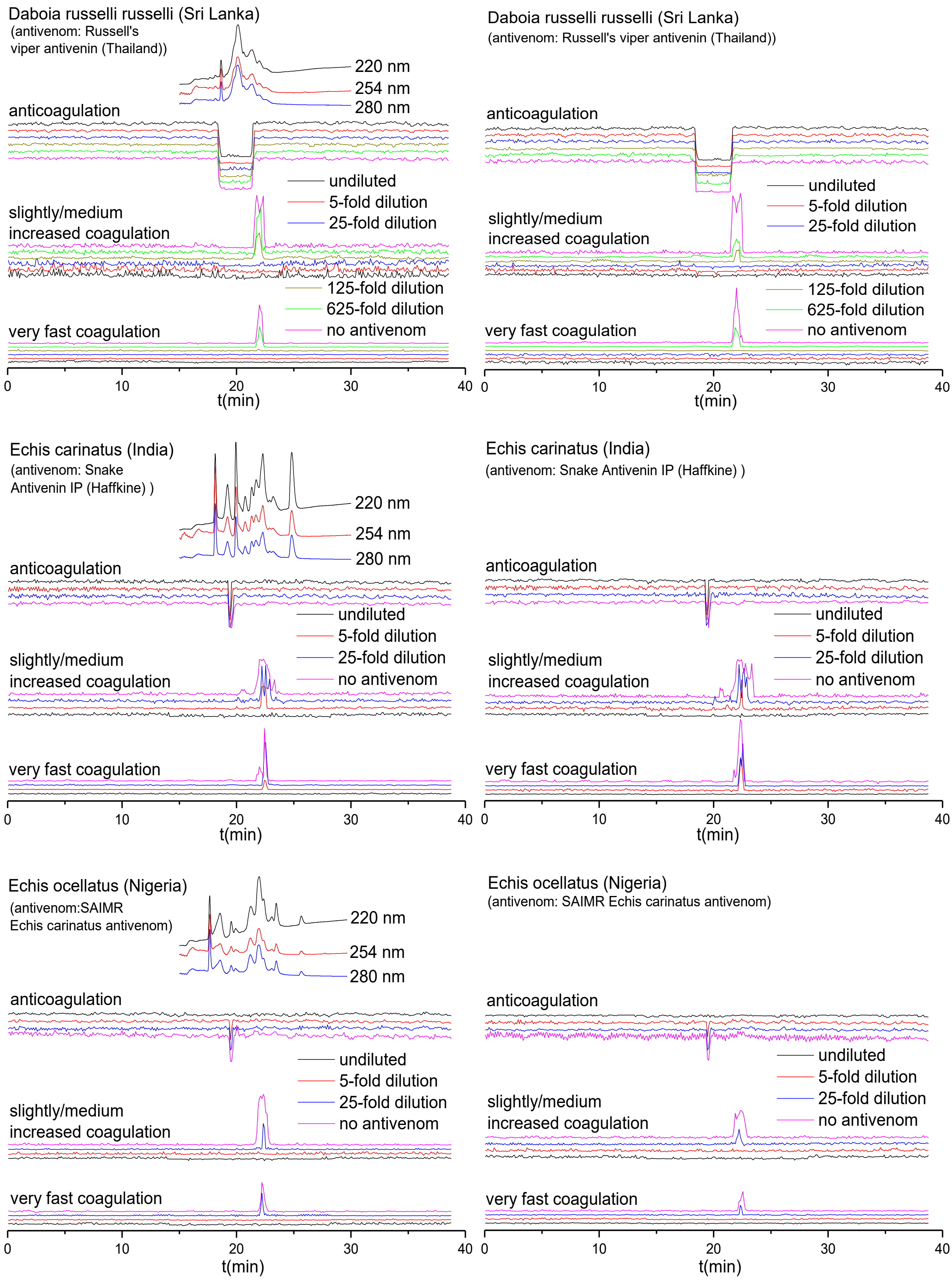

Figure S2. Duplicate chromatograms for antivenom neutralization of nanofractionated venom proteins involved in modulating plasma coagulation for *B. asper*, *C. rhodostoma*, *D. acutus*, *D. russelii*, *E. carinatus* and *E. ocellatus* venoms.

Duplicate analysis results showing neutralization by undiluted *Russell's viper* antivenom (Thailand) of *D. russelii* (Sri Lanka) venom nanofractionated at a 0.2 mg/ml concentration are depicted in Figure S3.

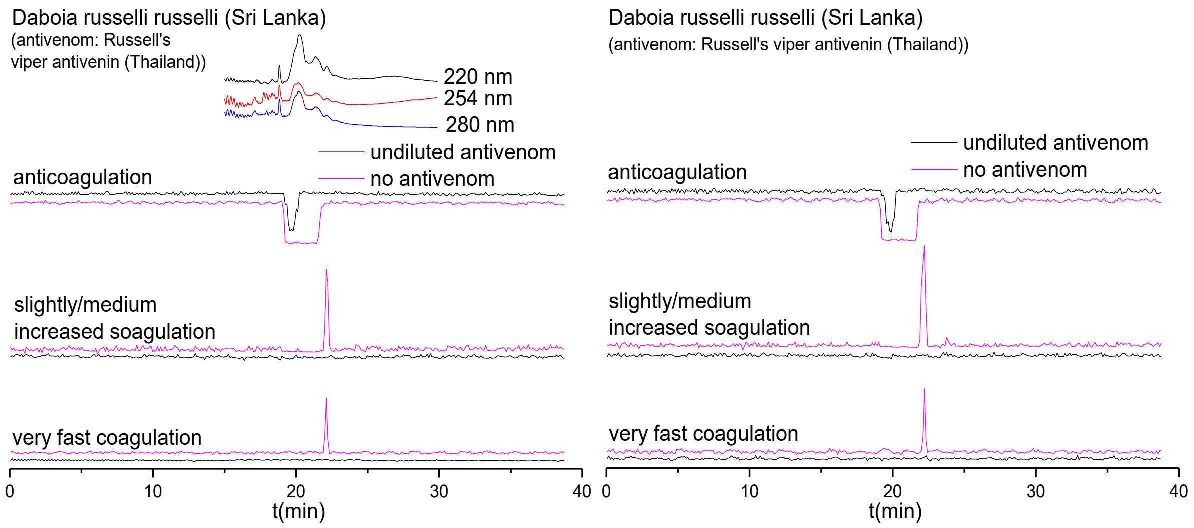

Figure S3. Duplicate chromatograms of 0.2 mg/ml nanofractionated *D. russelii* venom showing antivenom neutralization of venom proteins involved in modulating plasma coagulation (note: for all other antivenom experiments, 1.0 mg/ml venom concentrations were nanofractionated).

S3. Antivenom cross-reactivity

The results of duplicate analyses for antivenom cross-reactivity - SAIMR *Echis* antivenom *vs E. carinatus*, Snake Antivenin IP *vs E. ocellatus* and Snake Antivenin IP *vs D. russelii* - are shown in Figure S4. All venoms were nanofractionated at a concentration of 1.0 mg/ml. In the SAIMR *Echis* antivenom *vs E. carinatus* chromatograms, only the fast coagulation toxicities were fully neutralized by undiluted antivenom, while most of the positive peaks in the slightly/medium increased coagulation chromatogram were inhibited. The broad positive peak observed for the slightly/medium increased coagulation activity was reduced to a sharp positive peak, while the sharp but significant negative signal for the anticoagulation activity could not be neutralized at any antivenom concentration. By decreasing the antivenom concentration, the fast coagulation activity reappeared at the 5-fold antivenom dilution, while the weak and sharp positive peak observed in the venom-only analysis for fast coagulation was still fully neutralized by a 25-fold antivenom dilution. The activity of the sharp positive peak observed in the slightly/medium increased coagulation chromatogram in the undiluted antivenom analysis increased with decreasing antivenom concentrations. Conversely, both the first eluted and the last eluted sharp positive peaks present in the venom-only analysis for the slightly/medium increased coagulation activity were still fully neutralized by the 25-fold antivenom dilution.

In the Snake Antivenin IP *vs E. ocellatus* chromatograms, the Snake Antivenin IP was indeed able to neutralize the coagulant activity of *E. ocellatus* for which a clear concentration-dependence was observed. In the undiluted antivenom trace, no activity could be detected, implying that all measurable coagulopathic toxicities were fully neutralized. By decreasing the antivenom concentration, the coagulopathic activities started to appear, showing very weak activity for the 5-fold antivenom dilution, which increased at the 25-fold dilution.

When assessing the efficacy of Snake Antivenin IP *vs D. russelii* venom, all toxicities were fully neutralized by undiluted Snake Antivenin IP, as opposed to the antivenom analysis using *Russell's viper* antivenom (Thailand), where the procoagulant but not the anticoagulant toxicities were neutralized (as discussed above in section S2). Moreover, by decreasing the concentration of Snake Antivenin IP, the fast coagulation activity reappeared at the 125-fold antivenom dilution. For the slightly/medium increased coagulation profile, the peak intensity showed a clear concentration-dependence, for which only a weak positive peak appeared at the 5-fold antivenom dilution. The procoagulant peak intensity became stronger by further decreasing the antivenom concentration 25-fold and again when decreasing it 125-fold. Moreover, the broad negative anticoagulant peak became sharper and changed into several non-baseline separated negative peaks at the 5-fold antivenom dilution, while stronger negative peaks

were apparent at the 25-fold dilution. When using the 125-fold antivenom dilution, the results were similar to those of the venom-only analysis, implying that at this antivenom concentration no significant neutralization effects were observed.

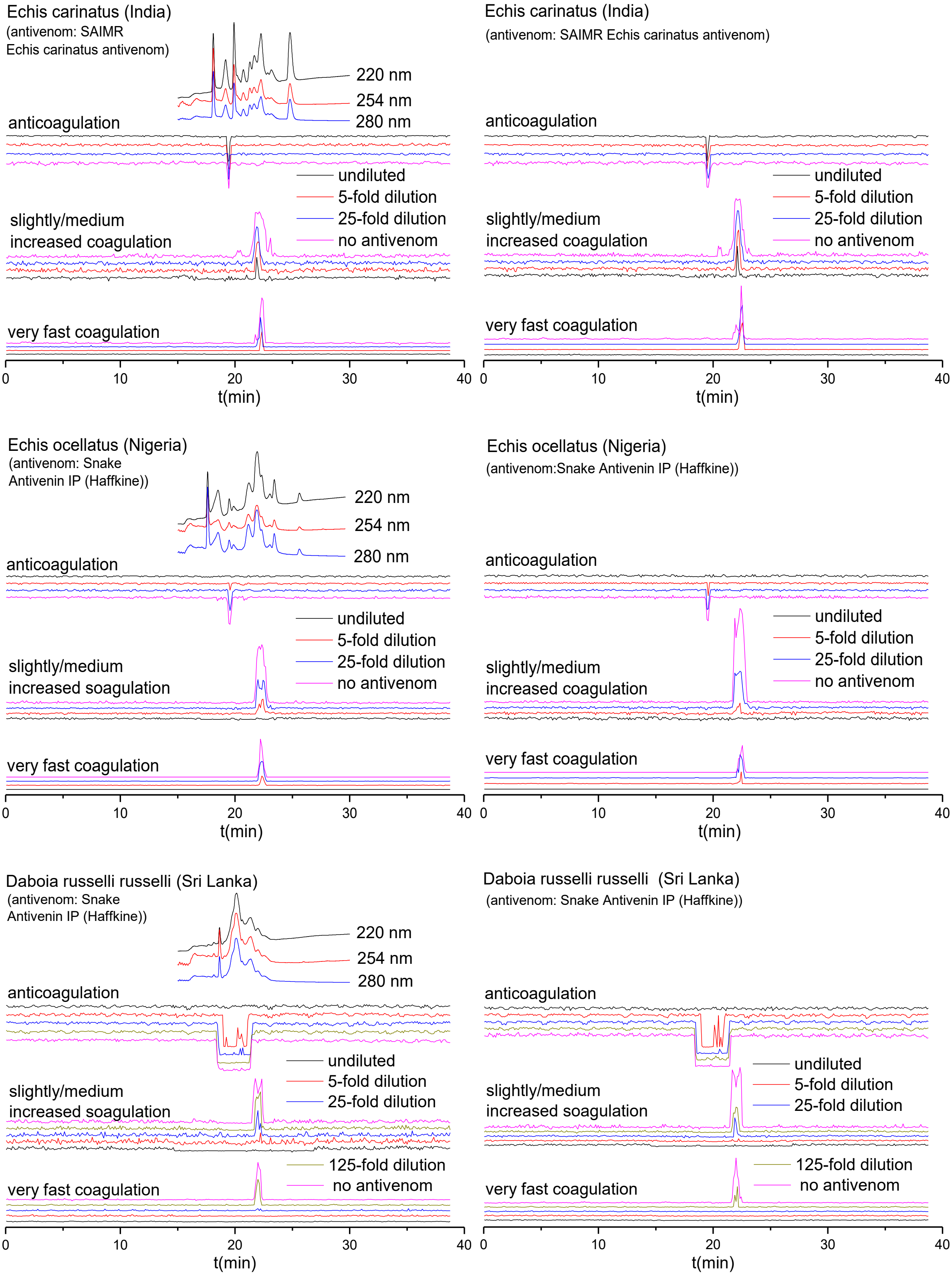

Figure S4. Duplicate bioassay chromatograms of evaluating antivenom cross-reactivity in neutralizing venom components involved in modulating plasma coagulation for SAIMR *Echis* antivenom *vs E. carinatus*, Snake Antivenin IP *vs E. ocellatus* and Snake Antivenin IP *vs D. russelii* .

S4 Coagulopathic properties of the Australian elapid venom *Oxyuranus scutellatus* and corresponding antivenom neutralization

Both the plasma coagulation modulating activities of *O. scutellatus* venom and the neutralization capability of Polyvalent Snake Antivenom (Australia-PNG) against nanofractionated *O. scutellatus* venom proteins were studied in a dose-response manner. The antivenom used to neutralize *O. scutellatus* venom, Polyvalent Snake Antivenom (Australia-PNG), was acquired from CSL Limited, Parkville, Victoria Australia. The duplicated reconstructed coagulation bioassay chromatograms are shown in Figures S5 and S6. In the venom activity chromatograms, a strong and broad anticoagulant peak was apparent, but no procoagulant peaks were observed at a concentration of 1.0 mg/ml. Slagboom *et al* did identify several procoagulant peaks while analyzing *O. scutellatus* venom, but analyzed this venom at a 5-fold higher concentration (i.e. 5.0 mg/ml venom) than the highest concentration tested in this study (i.e. 1.0 mg/ml venom) [1]. By decreasing the venom concentration, the strong and broad anticoagulant signals became narrower and sharper, splitting into multiple non-baseline negative peaks, but they were still apparent as a sharp negative peak at the 0.04 mg/ml venom concentration tested. No activity was observed anymore at the nanofractionation analysis of the low venom concentration of 0.008 mg/ml. In the presence of antivenom, the anticoagulant peaks were visualized as separate negative peaks, with increasing antivenom concentrations resulting in a reduction in the height of first cluster of negative peaks and eventually in their disappearance. Conversely, the second cluster of negative peaks was significantly reduced but not completely abolished, even in the presence of undiluted antivenom. From proteomics data retrieved from Slagboom et. al for *O. scutelatus* venom, VKT_OXYSC and VKT3_OXYSC, classified as the Kunitz-type serine protease inhibitor taicotoxin and scutellin-3, respectively, were identified as anticoagulant proteins. These are both serine protease inhibitors and VKT_OXYSC can inhibit factor Xa and α-factor XIIa. From the MS data, an *m/z* value of 788.1641^{9+} with a corresponding mass of 6993.2858 Da could be matched to VKT_OXYSC and an *m/z* value of 1091.8042^{6+} with a corresponding mass of 6536.8065 could be matched to VKT3_OXYSC. As these masses are in the range of the three-finger toxin family of proteins, it is likely that these toxins coeluted with other venom toxins that have anticoagulant properties. Moreover, PA2TA_OXYSC (PLA_2), PA2TB_OXYSC (PLA_2), PA21_OXYSC (PLA_2) and PA2TC_OXYSC (PLA_2) were also recovered from Mascot searches. These toxins are PLA_2s, but with no assigned anticoagulation activity. The PA2TA_OXYSC (PLA_2) and PA2TB_OXYSC (PLA_2) were reported as neurotoxins [2,3], and the PA2TA_OXYSC (PLA_2) can also catalyze the Ca^{2+}-dependent hydrolysis of 2-acyl groups in 3-sn-phosphoglycerides [2].

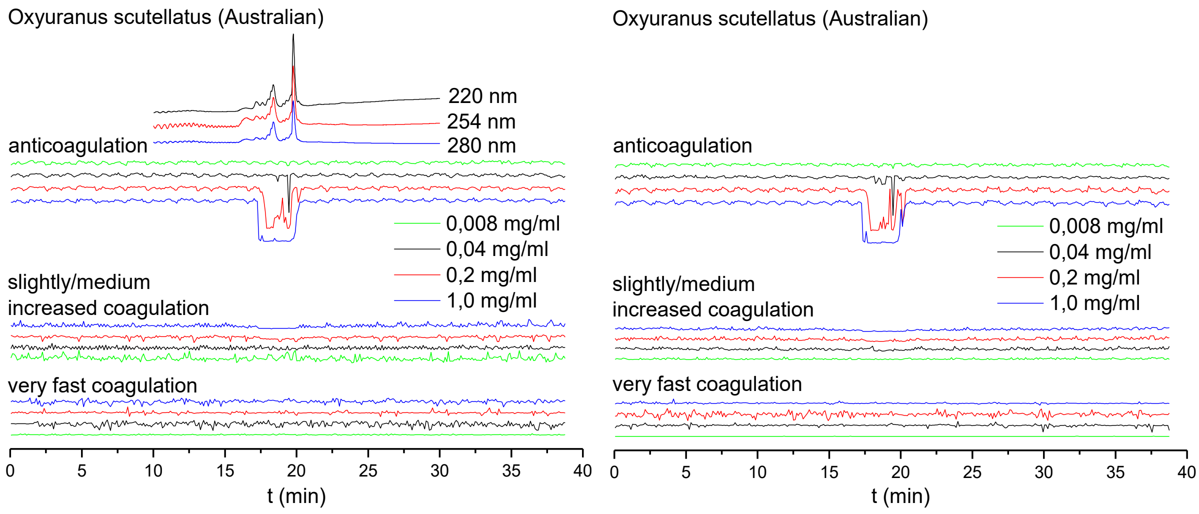

Figure S5. Reconstructed duplicate coagulation bioassay chromatograms of the *Elapid* venom of *O. scutellatus* after nanofractionation, analyzed at different concentrations.

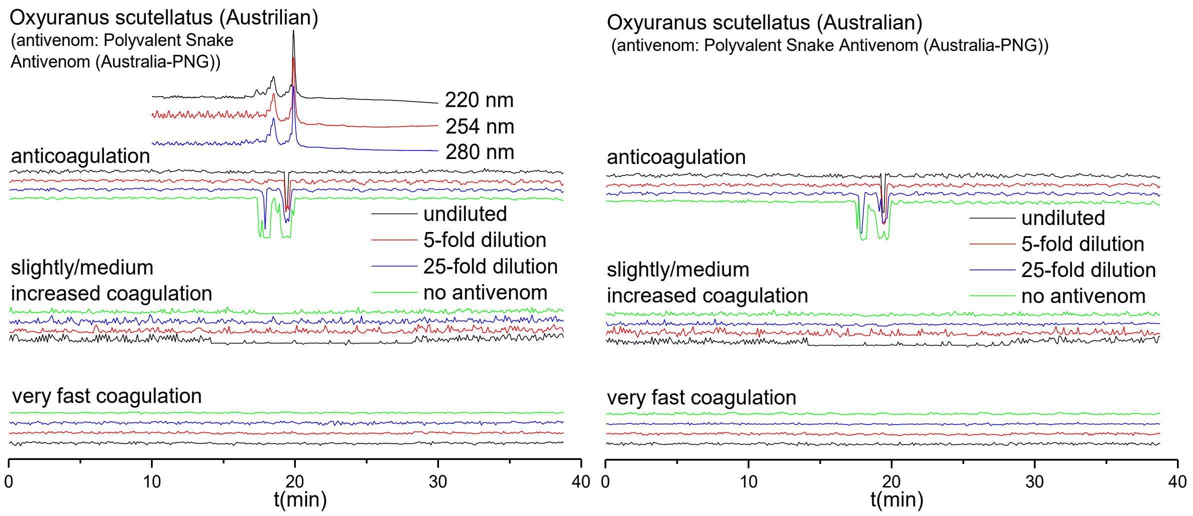

Figure S6. Duplicate bioassay chromatograms showing antivenom neutralization of nanofractionated *O. scutellatus* venom proteins involved in modulating plasma coagulation.

References

1. Slagboom, J.; Mladić, M.; Xie, C.; Vonk, F.; Somsen, G.W.; Casewell, N.R.; Kool, J. High throughput screening and identification of coagulopathic snake venom proteins and peptides using nanofractionation and proteomics approaches. *bioRxiv* **2019**, doi:10.1101/780155.
2. Cendron, L.; Mičetić, I.; Polverino de Laureto, P.; Paoli, M. Structural analysis of trimeric phospholipase A2 neurotoxin from the Australian taipan snake venom. *The FEBS journal* **2012**, *279*, 3121–3135.
3. Fohlman, J.; Eaker, D.; Karlsson, E.; Thesleff, S., Taipoxin, an extremely potent presynaptic neurotoxin from the venom of the Australian snake taipan (Oxyuranus s. scutellatus). *European journal of biochemistry* **1976**, *68*, 457–469.
